# The mechanism of α2-macroglobulin against oxidative stress and promoting cell proliferation in intervertebral disc degeneration

**DOI:** 10.1080/21655979.2021.2011638

**Published:** 2021-12-13

**Authors:** Hui Liang, Yuan Wang

**Affiliations:** a Department of Orthopaedic Surgery, The Second Hospital of Dalian Medical University, Dalian, China; b Department of Anesthesiology, Affiliated Zhongshan Hospital Dalian University, Dalian, Liaoning Province, China

**Keywords:** Α2-macroglobulin, intervertebral disc degeneration, nucleus pulposus cells, against oxidative stress, cell proliferation

## Abstract

This study was to explore mechanism of α2-macroglobulin (α2 MG) against oxidative stress and promote cell proliferation in the process of intervertebral disc degeneration (IDD). Nucleus pulposus cells extracted from the pathological tissues of IDD patients were treated with different concentrations of α2 MG (0, 0.1, 0.2, 0.4, 0.8, and 1 mg/mL), and were grouped into Group Z, Group A, Group B, Group C, Group D, and Group E, respectively. The cell proliferation, cell morphology, superoxide anion (SOA) content, and superoxide dismutase (SOD) activity were detected 1, 3, 5, and 7 days after the culture. The results showed that with the increase of α2 MG concentration and culture time, the O^2-^ content in nucleus pulposus cells gradually decreased (*P* < 0.05); the viability of the nucleus pulposus cells gradually increased after 1, 3, 5, and 7 days after culture, and the viabilities in the groups C, D, and E with α2 MG concentrations of 0.4 mg/mL, 0.8 mg/mL, and 1 mg/mL were much higher in contrast to viability in group Z (*P* < 0.05). Electrophoresis results showed that the relative expression of α-smooth muscle actin (α-SMA) protein in groups A, B, C, D, and E were 0.57, 0.66, 0.68, 0.77, and 0.84, respectively, all showing remarkable differences compared with the expression in group Z (*P* < 0.05). In summary, α2 MG can play a very good role in preventing oxidative stress and promoting cell proliferation in the process of IDD.

## 1. Introduction

Lumbar degeneration refers to a series of physiological and pathological changes such as lumbar bone hyperplasia, degeneration of the intervertebral disc, narrowing of the intervertebral space, compression of the lumbar vertebral ligaments, nerve roots, and soft tissue lesions [1]. Its clinical manifestations are mainly low back pain. The root cause may be the deterioration of body tissue quality and the damage caused by the increase in load caused by aging [2], and it may also be related to the pathological changes of the patient’s paraspinal muscles and endplates. Lumbar degeneration is mainly a physiological process that changes with age, but many factors will accelerate the aging and degeneration of the lumbar spine, causing a series of diseases and symptoms [3]. Common degenerative diseases of the lumbar spine include degeneration of the lumbar intervertebral disc fibrous annulus, degeneration of the nucleus pulposus of the intervertebral disc, degeneration of the lumbar vertebral body, degeneration of the lumbar facet joints, degeneration of the ligamentum flavum, degeneration of other ligaments, and fat infiltration of the paraspine muscles [4–7]. The intervertebral disc degeneration (IDD) refers to the loss of water from the intervertebral disc, which leads to the narrowing of the intervertebral space, and the degenerative changes of the lumbar intervertebral disc are the basic factor [8]. The degeneration of the nucleus pulposus is mainly manifested as a decrease in water content, and it can cause small-scale pathological changes such as instability and loosening of the vertebral segments due to water loss [9]. The current main treatment methods include surgical removal, conservative treatment, ozone ablation, collagenase dissolution, and artificial disc replacement [10].

At present, there are endless researches on the molecular pathogenesis of IDD, but there is no unified and commonly agreed answer. Many reports have mentioned that the pathogenesis of IDD is often accompanied by changes in related inflammatory regulatory factors in the body. In recent years, research on α2-macroglobulin (α2 MG) has continued to deepen, and it has been found that α2 MG has a wide range of diverse biological functions. Some studies have found that α2 MG can achieve anti-oxidation by lowering the content of cellular oxygen free radicals. α2 MG is the protein with the largest molecular weight in plasma, and it is synthesized by hepatocytes and mononuclear phagocyte system [11]. The molecular weight is about 652,000–800,000, composed of 4 subunits, and the sugar content is about 8%. It is closely related to the development and function of lymphoreticular cells [12,13]. α2 MG can be combined with a variety of molecules and ions, especially it can be combined with many proteolytic enzymes to affect the activity of these enzymes [14]. For example, it can combine with many endopeptidase enzymes (including serine, sulfhydryl, carboxyl proteolytic enzymes, and some metalloproteolytic enzymes) [15]. These proteolytic enzymes include plasmin, pepsin, chymotrypsin, trypsin, and cathepsin D. Studies have shown that the interaction of α2 MG with proteolytic enzymes can change the molecular conformation of α2 MG. When the enzyme is in a complex state, the active part of the enzyme is not inactivated, but it is not easy to act on the macromolecular substrate. It can be catalyzed and hydrolyzed by the α2 MG-protease complex to selectively protect the activity of certain proteases even in the presence of other anti-protease if the substrate is with a small molecular weight, which may be of great significance in immune reactions [16].

In summary, this study was developed to explore the role of A2M in preventing oxidative stress and promote cell proliferation in the process of IDD. It was hoped to process different concentrations of A2M on nucleus pulposus cells extracted from pathological samples of patients with IDD. In addition, this study determined the cell proliferation, cell viability, cell morphology, superoxide anion content, SOD viability value in different culture periods, and the appropriate concentration of A2M to treat nucleus pulposus cells. This study aimed to provide a scientific reference for the research of the mechanism of related diseases of IDD and the development of related drugs.

## 2. Materials and methods

### 2.1 Sources of test samples

The patients undergoing intervertebral disc herniation surgery in our Hospital in November 2020 were selected, and their nucleus pulposus excision part were collected as the research objects. A total of 8 samples were determined, including 5 males and 3 females. The patients were 23 ~ 48 years old, with an average age of 40.2 ± 4.32 years old. The excised parts of the nucleus pulposus of the specimens were all from segments L3 – L5. The Pfirmann lumbar disc herniation magnetic resonance imaging (MRI) grading of all specimens of the corresponding patient showed that the degree of disease was grade II.

**Figure 1.**
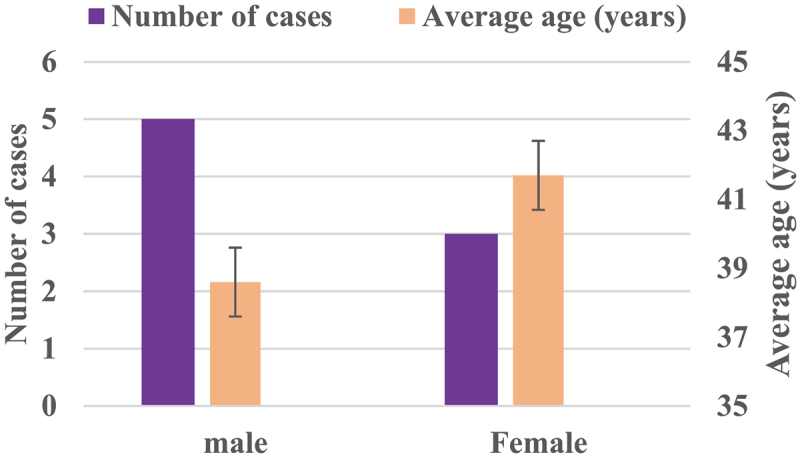
The average age and gender distribution of patients with nucleus pulposus removal.

**Figure 2.**
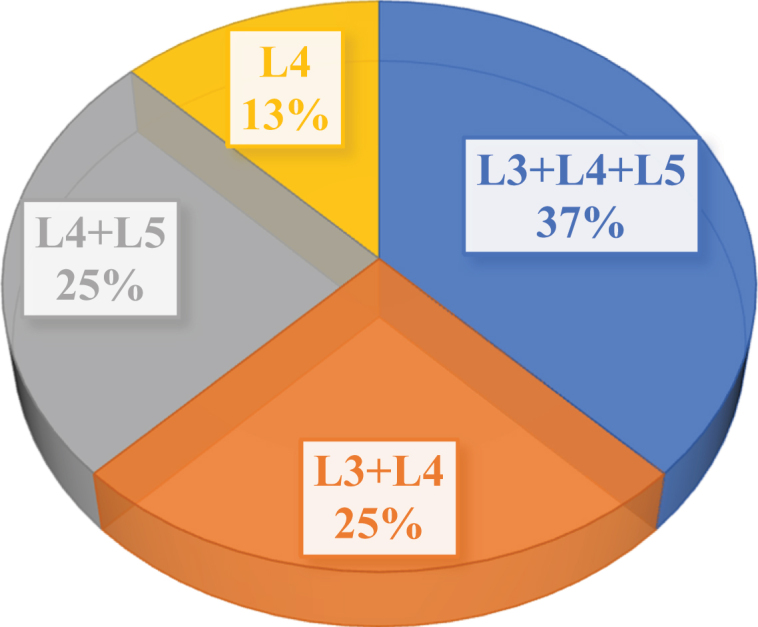
Distribution of nucleus pulposus removal site of patients.

Figure 1 showed the average age and gender distribution of patients undergoing nucleus pulposus removal surgery for intervertebral disc herniation. Figure 2 showed and the distribution of the nucleus pulposus removal sites of patients. Figure 1 illustrated that there were 5 males and 3 females in the enrolled nucleus pulposus patients in this study. The average age of male patients was 38.6 ± 5.4 years old, and that of female patients was 41.7 ± 4.8 years old, showing no significant difference (*P* < 0.05). As given in Figure 2, the source of the nucleus pulposus removal site of the 8 patients included 3 sites of L3+ L4+ L5, 2 sites of L3+ L4, 2 sites of +L4+ L5, and 1 site L4 area.

### 2.2 Experimental reagents and instruments

The experimental reagents adopted in this study included Dulbecco’s Modified Eagle Medium/Nutrient Mixture F-12 (DMEM/F12) 1:1 medium (Sigma, United States (US)), trypsin (Gibco, US), phosphate buffered saline (PBS) solution (Sigma), high-quality fetal bovine serum (FBS) (Sijiqing in Hangzhou of China), double antibody solution (Hyclone, US), vitamin C (Sigma), and α2 MG (ENZO, US).

The experimental instruments and consumables included disposable syringe filters (Pall, US), CO_2_ incubators (Heraeus HA50, Germany), clean benches (SANYO, Japan), inverted microscopes (Canon, Japan), electronic balance (Sartorius, Switzerland), electric thermostatic water bath (Shanghai Jinghong Test Equipment Co., Ltd.), 120 mesh nylon mesh filter (Beijing Medical Instrument Factory), ophthalmic surgical scissors (Shanghai Medical Instrument Factory), counting plate (Shanghai Qiujing Biochemical Reagent Instrument Co., Ltd.), autoclave (Japan Hirayama HA-50), 25 mL culture flask (Coring, US), SOD kit (Nanjing Jiancheng), super oxygen anion free radical (O_2_^−^) determination kit (Nanjing Jianshe), rabbit anti-human α-SMA polyclonal antibody and mouse anti-human glyceraldehyde-3-phosphate dehydrogenase (GAPDH) monoclonal antibody (Protein-tech), and goat anti-rabbit secondary antibody and goat anti-mouse secondary antibody (LinkTech).

### 2.3 Acquisition and cultivation of nucleus pulposus cells

The nucleus pulposus of the test substance was placed in a sterile laboratory environment. The nucleus pulposus tissues of the intervertebral discs from different sources were taken out and processed separately. Firstly, they were carefully cleaned with Hanks solution. When there was no blood stain, a 2 mm^3^ nucleus pulposus tissue was taken out for pathological examination. The remaining tissue was placed in a sterile tube (DMEM/F12), placed in an ice bucket, and transferred to the laboratory ultra-clean bench. Then, the tissue was separated using ophthalmic forceps to remove the surrounding connecting tissue and annulus fibrosus. Finally, the nucleus pulposus tissue for testing were selected carefully.

After that, the obtained nucleus pulposus tissue was washed twice with PBS and cut into pieces, and then digested with 0.25% trypsin at 37°C for 20 minutes. After the digestion was complete, the tissue was centrifuged at 1000 r/min for 5 minutes to remove the supernatant. Then, the cells were suspended in D-Hanks solution, mixed well, and centrifuged at 1000 r/min for about 5 minutes to remove the supernatant. Next, 2 mL FBS was added to dilute the cells. 1 mL of nucleus pulposus cell solution was taken out for trypan blue staining and counting the number of cells. The remaining nucleus pulposus cells were seeded in culture flasks at a density of 5 × 10^4^/mL for culture. When the cell growth reached 80%, subculture was carried out.

### 2.4 The effect of α2 MG on the proliferation and cell morphology of nucleus pulposus cells

A part of the nucleus pulposus cells after two passages were taken out and randomly divide into 6 parts, which were set as the control group (group Z), the experimental group 1 (group A), the experimental group 2 (group B), the experimental group 3 (group C), experimental group 4 (group D), and experimental group 5 (group E). They were transferred to 96-well plates for cultivation.

The α2 MG was dissolved with pre-cooled 0.01 mol/L PBS solution in advance, and configured into a 1 mg/mL α2 MG solution. After that, α2 MG was not added to group Z, and α2 MG of 0.1 mg/mL, 0.2 mg/mL, 0.4 mg/mL, 0.8 mg/mL, and 1 mg/mL was added to groups A, B, C, D, and E, respectively, and mixed evenly. The α2 MG was cultivated under the conditions of 5% CO_2_ and 37°C. 1, 3, 5, and 7 days after the culture, some cells were taken out of each group, and a part of each group was sampled and 10 ul of cholecystokinin 8 (CCK-8) reagent was added to each group. Another 2 hours later, it was measured in a microplate reader at 450 nm to measure the optical density (OD) values. Some samples were used for the morphology of nucleus pulposus cells in each period.

Since current studies have confirmed that the expression of CD24 in nucleus pulposus cells is significantly higher than that of other cells [17] and the expression of FBLN1 in nucleus pulposus cells in the degenerative phase is also significantly increased [18]. Therefore, this study would analyze the phenotype of the nucleus pulposus cells used in the experiment by detecting the expression of these two genes. Firstly, the RNA of the nucleus pulposus cells was extracted by Trizol extraction method, and then the RNA was reverse-transcribed into cDNA using Takara kit. Then, the CD24 and FBLN1 gene sequences were amplified by fluorescence quantitative PCR, using Actin β as the internal reference. The above experiment should be repeated three times to collect data and analyze the results.

### 2.5 Detection on superoxide anion (SOA) content, and superoxide dismutase (SOD) activity

The SOA free radical detection kit was adopted to detect the SOA content in nucleus pulposus cells in different culture stages. The specific experimental procedures were strictly referred to the instructions for the SOA free radical detection kit. In addition, the SOD determination kit was used in this study. Before the SOD activity was detected, it was necessary to take the nucleus pulposus cells in the logarithmic growth phase in each group, which were digested with 2.5 gL of trypsin to pipette to a cell suspension of 1 × 10^6^/mL. Then, the cells were placed in the refrigerator at −80°C to freeze for 4 hours. After that, the cells were melt and pipetted again to form a cell suspension. This process was repeated 3 times. Then, the lysate was aspirated and centrifuged at 2000 r/min for 10 minutes to separate the supernatant, which was filtered with a 0.45 um filter membrane for use. 10 μL of lysate was adopted to determine the protein concentration of each group of samples using the bicinchoninic acid (BCA) method to calculate the SOD activity per mg of protein.

### 2.6 Western blotting method to detect the expression of the target protein

In this study, Western blot was used to detect the protein expression of the target protein α-SMA in each group of nucleus pulposus cells after 7 days of culture. Firstly, the portion of cells was taken from each group, added with the cell culture medium, and washed with PBS 3 times. The Radio Immunoprecipitation Assay (RIPA) lysis solution was adopted to extract the total cell protein, and the BCA method was applied to determine and balance the concentration of each histone. Afterward, each group of samples was subjected to sodium dodecyl sulfate polyacrylamide gel electrophoresis (SDS-PAGE), and the electrophoresis was maintained at a constant pressure of 120 V for 1.5 hours. After the electrophoresis, the membrane transfer operation was performed, and the protein was transferred to the polyvinylidene fluoride (PVDF) membrane with a constant current of 280 mA for about 90 minutes. Then, it was blocked with 5% skimmed milk powder solution for 1 hour, and then incubated with the primary antibody against human α-SMA (Proteintech, US) and cultured with mouse anti-human GAPD (Proteintech, US) overnight. The next day, the membrane was washed with Tris Buffer Solution T (TBST) 3 times, and then incubated with goat anti-rabbit secondary antibody (MultiSciences) and goat anti-mouse secondary antibody (MultiSciences) for 1 hour. The smooth muscle actin antibody used in this process was a monoclonal mouse anti-human antibody. After the membrane was washed 3 times with TBST, it was developed by chemiluminescence method. The gray value of the band was analyzed using the image software, and the calculation equation of the target protein expression (*D*) was shown as follows:

(1) $$D=\frac{H}{G}$$

In the above equation (1), H represented the net gray value of the target protein α-SMA, and G represented the net gray value of GAPDH.

### 2.7 Statistical analysis

The test data was processed using SPSS19.0 statistical software. The measurement data was expressed in the form of mean ± standard deviation ($\bar{x}$ ± s) or median and lower quartile and upper quartile form, and the comparison of the mean between each group was by Mann Whitney U test. The count data was expressed by percentage (%), and the χ^2^ test was used. *P* < 0.05 indicated that the difference was statistically significant.

## 3. Results

### 3.1 Morphology of nucleus pulposus cells

**Figure 3.**
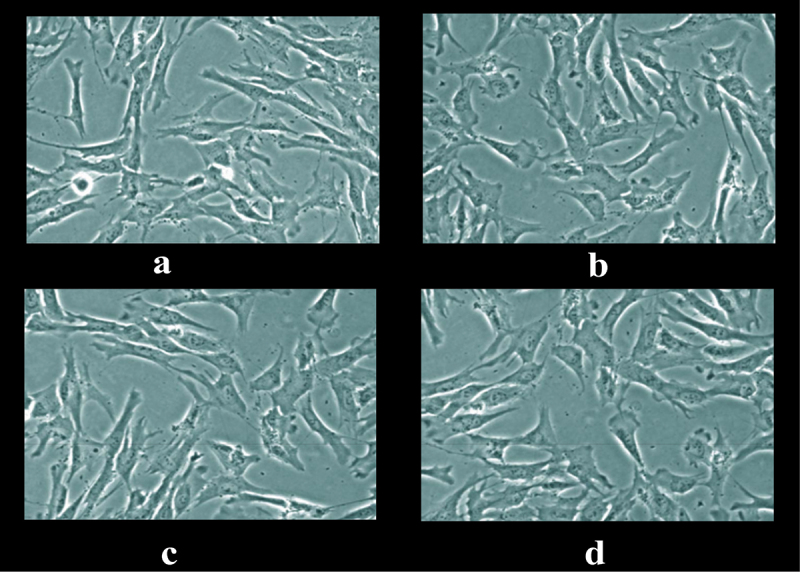
Microscopic image of nucleus pulposus cells in the first transfer generation.

Note: Figures a, b, c, and d were the images of nucleus pulposus cells taken from the L3+ L4+ L5 site, L3+ L4 site, L4+ L5 site, and L4 site alone, respectively.

Figure 3 was a microscopic image of subcultured nucleus pulposus cells extracted and cultured from the nucleus pulposus tissue. It showed that in the microscopic image of nucleus pulposus cells, the shape was the same, most of them were fusiform, and a few were similar to round, no matter where the tissues were extracted. The protrusions of the spindle cells were emitted along the long axis of the cell body, and most of the cells showed relatively thick protrusions, with 1 protrusion at each pole. A small number of cells showed multiple protrusions, the round-like cells were similar in appearance to chondrocytes, and the protrusions were emitted from the periphery of the cell body and were dendritic.

### 3.2 Phenotypic analysis of nucleus pulposus cells

**Figure 4.**
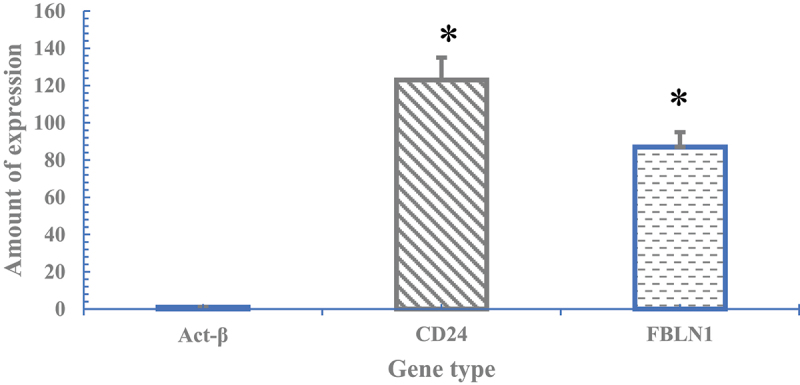
CD24 and FBLN1 gene expression results in myeloid cells.

Figure 4 showed the CD24 and FBLN1 gene expression results of nucleus pulposus cells sampled. The results showed that in the nucleus pulposus cell samples used in this study, the expression of CD24 and FBLN1 genes were significantly increased compared with the expression of Act-β, and the differences were statistically significant (*P* < 0.05).

### 3.3 Changes in the number and morphology of nucleus pulposus cells in each culture period

**Figure 5.**
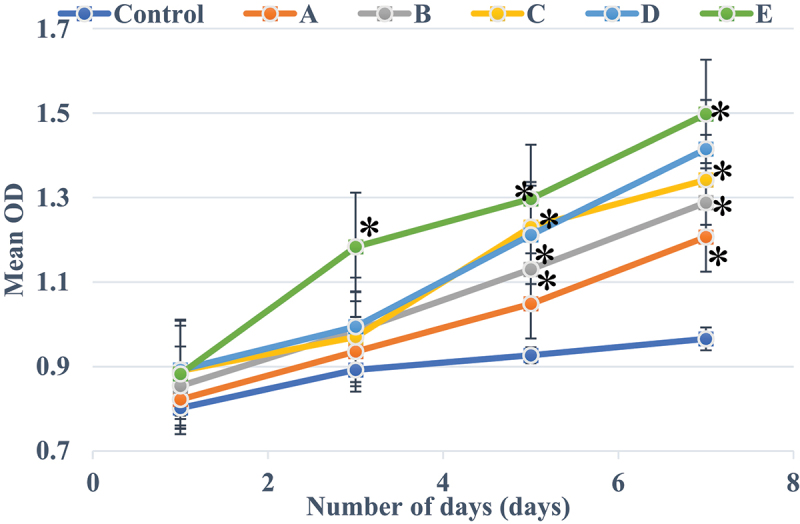
CCK-8 OD values of nucleus pulposus cells with different concentrations of α2 MG in each period of culture.

Note: * meant the difference was statistically great in contrast to the CCK-8 OD value of patients in the Con group (*P* < 0.05).

Figure 5 showed the growth curve of nucleus pulposus cells in each culture period. The growth rate of nucleus pulposus cells was greatly different under various α2 MG concentrations. Among them, the average OD values of the control group were 0.8020 ± 0.0186 (group Z, average value, time of repetition (TR) = 6), 0.8921 ± 0.0457 (group Z, average, TR = 6), 0.9267 ± 0.0302 (group Z, average, TR = 6), and 0.9654 ± 0.0316 (group Z, average, TR = 6) after 1, 3, 5, and 7 days of culture, respectively, showing no statistical differences (*P* > 0.05). The OD values of nucleus pulposus cells in groups A, B, C, D, and E were much greater than that of group Z after culture for 5 and 7 days, and the differences were statistically remarkable (*P* < 0.05). Among the measured OD values of each group after 3 days of culture, only the group E showed an obviously higher OD value than the group Z with statistically great difference (*P* < 0.05), and the others showed no visible difference (*P* > 0.05).

**Figure 6.**
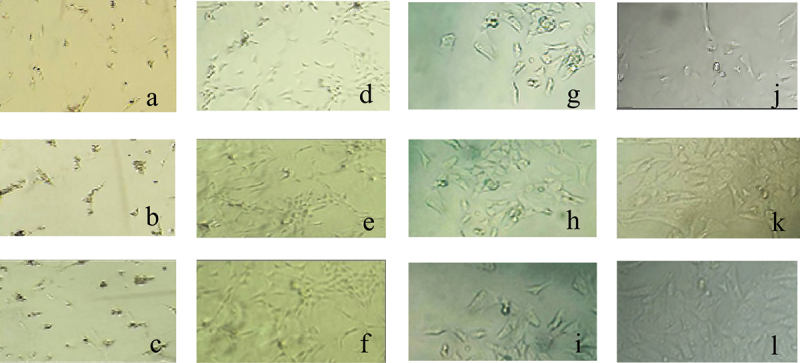
The morphology of nucleus pulposus cells in each culture period.

Note: the first row was the cell diagram of group Z, and the second row showed the cell diagram of group D; a, b, and c were all nucleus pulposus cells observed after 1 day; d, e, and f were all nucleus pulposus cells observed after 3 days; g, h, and j were all nucleus pulposus cells observed after 5 days; and k, l, and m were all nucleus pulposus cells observed after 7 days.

Figure 6 showed the morphology of nucleus pulposus cells in each culture period. With the increase of culture time, the number of nucleus pulposus cells in each group increased dramatically. At the same time, nucleus pulposus cells gradually adhered to the wall during the culture process. When they just adhered to the wall, nucleus pulposus cells were short fusiform and polygonal. With the extension of the culture time, the cytoplasm of nucleus pulposus cells gradually extended outwards, forming part of the protrusions, and then the protrusions gradually elongated, and the nucleus pulposus cells gradually became fusiform in shape.

### 3.4 Changes in SOA content and SOD activity of nucleus pulposus cells in each culture period

**Figure 7.**
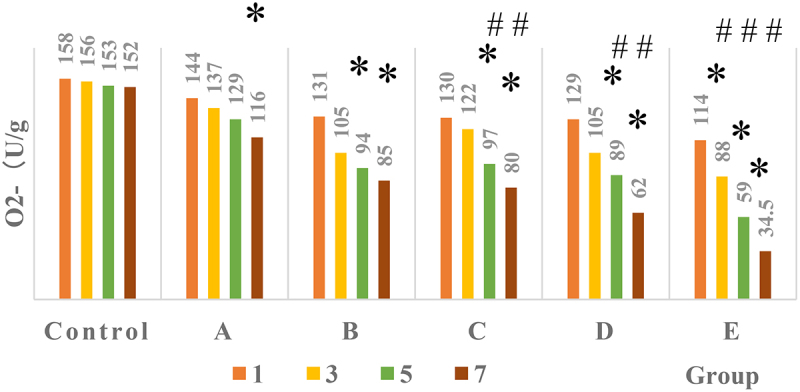
Comparison on SOA content in each group of patients.

Note: * indicated that the SOA content of each group sample was remarkably different from the group Z (*P* < 0.05); # indicated that the SOA content of each group sample showed a visible difference within the group (*P* < 0.05).

Figure 7 illustrated the distribution of SOA (O^2-^) content in nucleus pulposus cells during each culture period. It suggested that under different α2 MG concentrations, the O^2-^ content of nucleus pulposus cells in each culture stage changed to a certain extent. The SOA in nucleus pulposus cells of the control group (group Z) after 1d, 3d, 5d, and 7d of culture were 158, 156, 153, and 152 U/g (group Z, average values, TR = 6), respectively. The O^2-^ contents in group A after 1 day, 3 days, 5 days, and 7 days of culture were 144, 137, 129, and 116 U/g (average values, TR = 6), respectively; those in group B were 131, 105, 94, and 85 U/g (average values, TR = 6), respectively; those in group C were 130, 122, 97, and 80 U/g (average values, TR = 6), respectively; those in group D were 129, 105, 89, and 62 U/g (average values, TR = 6), respectively; and those in group E were 114, 88, 59, and 34.5 U/g (average value, TR = 6), respectively. The O^2-^ content in nucleus pulposus cells cultured for 7 days in group A was obviously lower than the content in group Z (*P* < 0.05); that cultured for 5 and 7 days in groups B, C, and D was much lower in contrast to the group Z (*P* < 0.05); and that cultured for 3, 5, and 7 days in group E was lower obviously than compared with the control Z (P < 0.05). In addition, the O^2-^ content of groups C and D decreased greatly after 5 and 7 days of culture compared with the 1 day of culture (*P* < 0.05), and that in group E decreased dramatically after 3, 5, and 7 days of culture compared with the 1 day of culture (*P* < 0.05).

**Figure 8.**
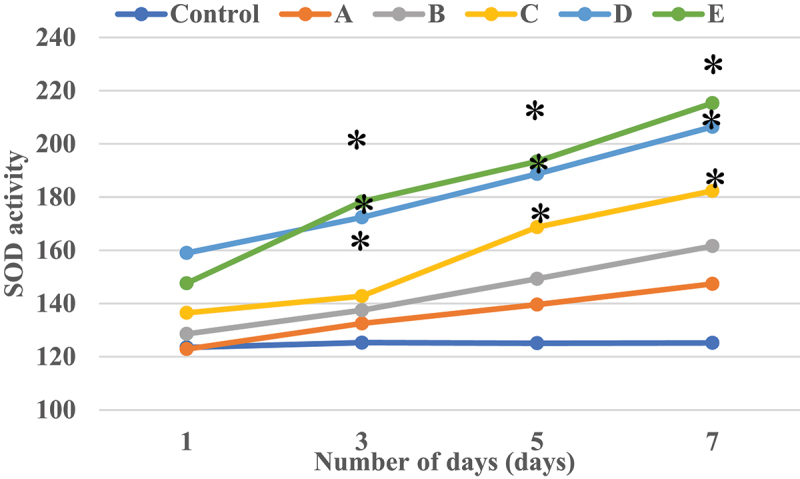
The SOD activity curve of nucleus pulposus cells in each culture period.

Note: * marked the obvious different in SOD activity in contrast to the group Z (*P* < 0.05).

Figure 8 illustrated the SOD activity curve of nucleus pulposus cells in each culture period. It revealed that the SOD activity of nucleus pulposus cells was notably different under different α2 MG concentrations. With the increase of α2 MG concentration, the SOD activity of nucleus pulposus cells gradually increased after culture for 1 day, 3 days, 5 days, and 7 days. When the α2 MG concentration was 0.4 mg/mL, 0.8 mg/mL, 1 mg/mL, the SOD activities of cells in groups C, D, and E were dramatically higher than the SOD activity of the group Z, and the differences were statistically remarkable (*P* < 0.05).

### 3.5 Test results of target protein in each group of samples

**Figure 9.**
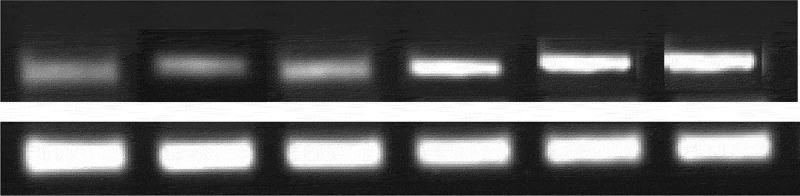
The electrophoresis results of each group of samples.

Note: the upper part of the electrophoresis band in the figure was the electrophoresis result of the target protein α-SMA, and the lower part was the electrophoresis result of the reference protein GAPDH. The images from left to right were the electrophoresis results of groups A, B, C, D, E, and Z, respectively.

**Figure 10.**
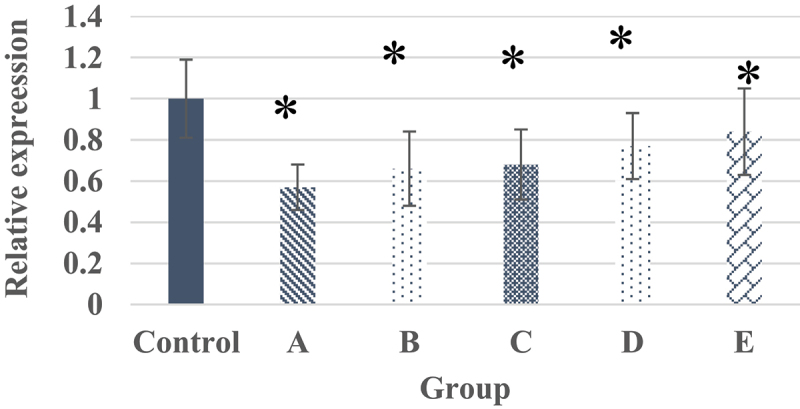
The target protein content of each group of samples.

Note: * marked the obvious different in relative expression of target protein in contrast to the group Z (*P* < 0.05).

Figures 9 and 10 were the electrophoresis results of each group of samples and the target protein content distribution diagram of each group of samples, respectively. After 7 days of culture of nucleus pulposus cells in each group, the electrophoretic band of the target protein gradually deepened as the concentration of α2 MG continues to increased. At different concentrations of α2 MG, the relative expression of α-SMA protein in nucleus pulposus cells of each group was memorably different, and the relative expression of α-SMA protein in groups A, B, C, D, and E were 0.57, 0.66, 0.68, 0.77, and 0.84 (average values, TR = 6), respectively, with statistical significance in contrast to the expression in group Z (*P* < 0.05). In addition, the expression of the target protein gradually increased as the concentration of α2 MG continues to increased.

## 4. Discussion

At present, the specific pathogenesis of the academic community has not yet come to the commonly agreed answer, and which type of tissue composition or chemical factor in the nucleus pulposus tissue causes the inflammatory response is not completely determined [19]. However, the current research generally believes that the existence and activities of phospholipase A2 (PLA2), prostaglandin carbon monoxide, immunoglobulin, etc [20]. Recent studies have also pointed out that as a broad-spectrum protease inhibitor, α2 MG plays an anti-inflammatory and self-defense mechanism similar to white blood cells in the body [21]. In the early stage of articular cartilage degradation, a large amount of α2 MG is secreted in the joint cavity to counter the damage of matrix metalloproteinases and other inflammatory factors to cartilage [22]. However, its own secretion volume may be extremely limited. The secretion volume drops sharply one week after injury, which is not enough to fight the inflammatory factors that initiate cartilage destruction for a long time. This suggests the importance of supplementing α2 MG in the early onset of post-traumatic osteoarthritis [23]. Based on this, different concentrations of α2 MG were applied to treat nucleus pulposus cells extracted from IDD patients in this study. It was hoped to explore the effects of α2 MG on the process of IDD against oxidative stress and promoting cell proliferation by measuring cell proliferation, cell viability, cell morphology, SOA content, and SOD viability value in different culture periods.

In the microscopic image of nucleus pulposus cells, the shape was the same, most of them were fusiform, and a few were similar to round, no matter where the tissues were extracted. However, the results obtained may have certain limitations due to the low number of samples of various tissues in this study. The growth curve of each group of samples at each culture stage showed that the OD values of nucleus pulposus cells in groups A, B, C, D, and E were obviously greater than the OD values in group Z after culture for 5 days and 7 days, with statistically significant differences (*P* < 0.05); the OD value of the E group 3 days after culture was observably higher compared to the control Z only (*P* < 0.05), and the rest were not greatly different (*P* > 0.05). Such results suggest that the number of nucleus pulposus cells has increased dramatically with the increase of α2 MG concentration and incubation time. Among them, the α2 MG concentration is in the range of 0.1 ~ 1 mg/mL. As the concentration increases, the cell proliferation is faster, which means that α2 MG exerts the effect of promoting the proliferation of nucleus pulposus cells. The measurement results of O_2_^−^ content in nucleus pulposus cells during each culture period showed that with the increase of α2 MG concentration and culture time, the O_2_^−^ content in nucleus pulposus cells gradually decreased (*P* < 0.05). It indicates that α2 MG is conductive to scavenging oxygen free radicals, and can play a role against oxidative stress to a certain extent. The SOD cell viability curve of nucleus pulposus cells showed that the viability of nucleus pulposus cells after 1d, 3d, 5d, and 7d of cultures gradually increased with the increase of α2 MG concentration. The cell viability of groups C, D, and E with α2 MG concentrations of 0.4 mg/mL, 0.8 mg/mL, and 1 mg/mL was memorably higher than that of group Z, and the differences were statistically visible (*P* < 0.05). Such results reveal that α2 MG can inhibit the apoptosis of IDD nucleus pulposus cells, and enhance the vitality of nucleus pulposus cells, which are consistent with the findings of Wang et al. (2017) [24]. The electrophoresis results of each group of samples and the target protein content distribution results of each group of samples showed that after 7 days of culture, the electrophoretic bands of the target protein gradually deepened as the concentration of α2 MG in each group of nucleus pulposus cells continued to increased. Under different concentrations of α2 MG, the relative expression of α-SMA protein in nucleus pulposus cells of each group was signally different; the relative expression of α-SMA protein in groups A, B, C, D, and E were 0.57, 0.66, 0.68, 0.77, and 0.84, respectively, and there were significant differences statistically in contrast to the group Z (*P* < 0.05).

In summary, the results of this study confirmed the anti-oxidative stress and cell proliferation effects of α2 MG in intervertebral disc degeneration. The mechanism may lie in the anti-inflammatory and self-defense mechanism of α2 MG, but the specific action process needs further in-depth study. However, there were still some shortcomings in this study. When the effect of α2 MG on nucleus pulposus cells was explored, the experimental samples drawn were from a single source and a small number, which may lead to systematic errors in the experimental results. In addition, there were few test parameters about the effect of α2 MG on nucleus pulposus cells in this study, and the conclusions that can be drawn were relatively limited, and further analysis was needed in subsequent studies.

## 5. Conclusion

In this study, different concentrations of α2 MG were used to treat nucleus pulposus cells extracted from patients with IDD. The results showed that α2 MG cou promote the cell proliferation of degenerated nucleus pulposus cells, enhance the viability of nucleus pulposus cells, reduce the content of superoxide anions in the cells, and increase the activity of SOD. In addition, it can resist oxidative stress and promote cell proliferation during the IDD. In summary, α2-macroglobulin could play the roles of anti-oxidative stress and promoting cell proliferation in IDD, which provided a new research direction for the clinical diagnosis and treatment of patients with intervertebral disc herniation.

## Highlights

The echanism of α2MG in IDD was explored.

The concentration range of α2MG suitable for nucleus pulposus cells was determined.

α2MG can promote the proliferation and anti-oxidation of nucleus pulposus cells.
